# Impact of size and fragmentation of the anteroinferior glenoid rim on clinical and functional outcomes of non-operatively treated Bony Bankart lesions in middle-aged population

**DOI:** 10.1007/s00402-024-05466-4

**Published:** 2024-08-06

**Authors:** Gregorio Secci, Francesco Lazzarini, Marco Distefano, Tommaso Porciatti, Filippo Tonelli, Marco Mugnaini, Luigi Zanna

**Affiliations:** 1 Department of Orthopedic Surgery, Santa Maria Annunziata Hospital, ASL Toscana Centro - Via Antella 58, 50012 Bagno a Ripoli, Italy; 2 Department of Shoulder Surgery, AOU Careggi, University Hospital of Florence, Florence, Italy; 3 University of Florence, Florence, Italy

**Keywords:** Glenoid rim fracture, Shoulder dislocation, Non-operative treatment, Fragment size, Rim fragmentation

## Abstract

**Introduction:**

The optimal treatment approach for Bony Bankart remains a subject of considerable debate among shoulder surgeons. Existing literature highlights low recurrence rates and high patient satisfaction with nonoperative treatment, particularly in the middle-aged population. This study aimed to evaluate the recurrence rate of dislocation, as well as the clinical and functional outcomes in middle-aged individuals treated nonoperatively following an acute bony Bankart fracture. Additionally, the impact of glenoid rim size and fragmentation on the treatment outcome was investigated.

**Material and methods:**

A prospective analysis was conducted on 20 patients aged over 50 with nonoperatively treated bony Bankart fractures, ensuring a minimum follow-up of 24 months. The study population was categorized based on fragment size (small and medium) according to Kim classification and glenoid rim fragmentation (type 1b and 1c) according to Scheibel classification. Data including UCLA score, Rowe score, recurrence rate, clinical instability, and range of motion (ROM) were collected and analyzed.

**Results:**

The average UCLA and Rowe scores were 32.15 ± 2.85 and 93.85 ± 2.19, respectively, with no instances of dislocation recurrence. The affected shoulder exhibited no significant reductions in ROM compared to the contralateral side, except for a loss of external rotation (ER) (13.08° ± 7.51; *p* = 0.005). No differences were observed based on fragment size, although patients with multifragmented glenoid rims showed a greater loss of ER compared to those with a solitary fragment, albeit not reaching statistical significance.

**Conclusion:**

Nonoperative treatment appears to be a viable and effective option for middle-aged individuals with bony Bankart fractures, resulting in favorable functional outcomes and a low risk of recurrence. Additionally, a notable loss of external rotation was observed in fractures with glenoid rim fragmentation.

**Level of evidence:**

IV.

## Introduction

Bony Bankart fractures are fractures of the anteroinferior glenoid rim associated with shoulder dislocation [1–3]. These fractures, present in 4–70% of all the anteroinferior glenohumeral dislocation [1, 4, 5], are more prevalent in males and middle-aged patients [6, 7]. Conversely, in younger individuals, labrum lesions or isolated osteochondral avulsions are typical [4]. Bony Bankart fractures often coexist with other injuries, with traumatic rotator cuff tears being the most common, particularly in the middle-aged population [8]. Bigliani et al. first classified glenoid rim fractures in 1998, categorizing them into three groups based on radiographic evaluation [5] Recently, new classifications have emerged, considering both the size of the bone defect [9] and fragment characteristics [10, 11], using more precise diagnostic tools such as CT scans and MRI.

The optimal treatment for anteroinferior glenoid rim fractures remains a contentious issue among shoulder surgeons [1, 4, 11–14]. Several studies suggest that bony Bankart fractures play a crucial role in the development of recurrent shoulder instability [7, 15, 16], prompting many authors to advocate surgical intervention. Both open and arthroscopic procedures have been proposed [1, 17–24], yet a universally accepted gold standard for treatment remains elusive [17]. Conversely, some authors have reported low recurrence rates and high patient satisfaction following nonoperative treatment, especially in the middle-aged population [4, 12, 13, 25, 26].

The aims of this study were (1) to analyze the recurrence rate of dislocation following nonoperative treatment of acute bony Bankart fractures in the middle-aged population and (2) to assess clinical and functional outcomes at a minimum 24-month follow-up using the Rowe score, UCLA score, and Range of Motion (ROM). Additionally, we aimed to compare clinical scores between small-sized and medium-sized lesions and between single fragment and multifragmented glenoid rim fractures.

We hypothesize that nonoperative treatment may be a successful option for individuals over 50 years old in terms of residual instability and shoulder function recovery, with no discernible differences based on the size or quality of the fragment.

## Materials and methods

**Fig. 1:**
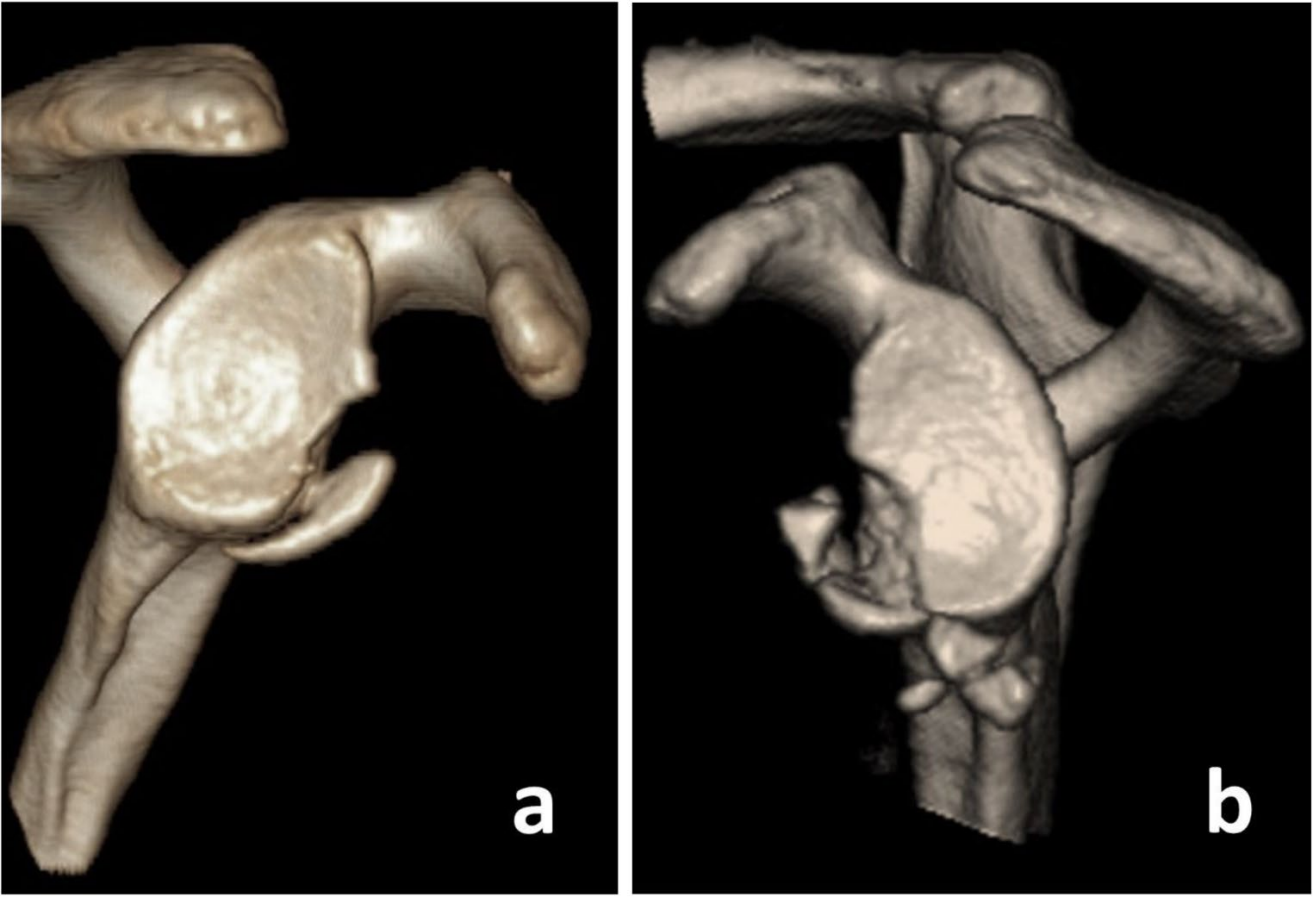
**a** Scheibel type 1b: solitary bony fragment; **b** Scheibel type 1c: multifragmented rim fracture, with a fragment placed in the axillary pouch

**Fig. 2:**
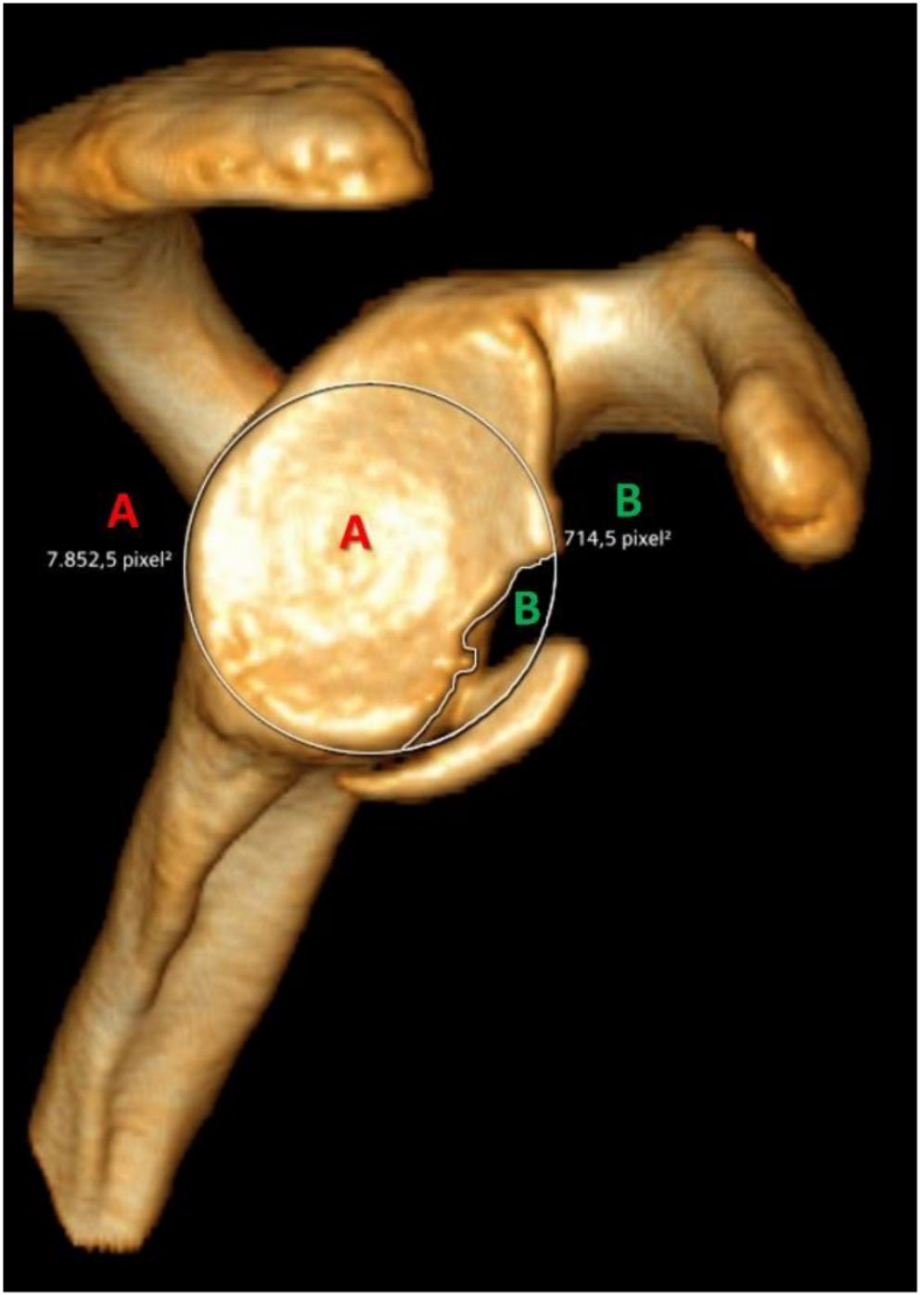
Three-dimensionally reconstructed computer tomography “en-face” view of the glenoid surface with a bony Bankart lesion. Though the software Syngo.via webviewer (Siemens Healthineers, Erlangen, Germany), according to the “best-fit circle” method [27], the glenoid area was measured (A = 7852,5 pixel^2^), and the bone loss was marked (B = 714,5 pixel^2^). So, the bone loss size was calculated as A/B (7852,5/714,5 = 10,99%)

A prospective analysis was conducted on 20 patients who experienced traumatic anterior shoulder dislocation associated with a nonoperatively anteroinferior glenoid margin fracture from May 2018 to March 2021. We included consecutively enrolled patient over 50 years old with fracture of the anteroinferior margin of the glenoid classified as type 1 according to the Scheibel Classification [10, 11][Table 1][Fig. 1], measured as small and medium size according to the Kim Classification [9] [Table 1], and patient with a minimum follow-up of 24 months. Patients with the humeral head not centered after reduction, those with concomitant shoulder lesions potentially affecting post-dislocation recovery (such as brachial plexus damage, coracoid fractures, etc.), patients with a history of shoulder surgery, and Off-Track Hill-Sachs (HS) lesions according to Di Giacomo et al. [16]. were excluded. Glenoid bone loss was quantified using the “best-fit circle method” according to Sugaya et al. [27]. , employing the Syngo via web viewer software (Siemens Healthineers, Erlangen, Germany) [Fig. 2].

**Table 1:** Glenoid defects classifications

		Quantitative classification of the fragment					
***Kim et al.*** [9]	*Small-sized*	< 12.5%					
	*Medium-sized*	> 12.5% and < 25%					
	*Large-sized*	> 25%					
		**Qualitative classification of the fragment**					
***Scheibel et al.*** [10, 11]	*Type 1*	*Acute* fragment type-lesion					
			*1a*	Osteochondral avulsion lesion			
			*1b*	Single fragment rim fracture			
			*1c*	Multifragmented rim fracture			
	*Type 2*	*Chronic* fragment-type lesion					
				Malunited fragment			
	*Type 3*	*Chronic* bone loss without fragment					
			*3a*	bone loss < 25% (of the glenoid surface)			
			*3b*	bone loss > 25% (of the glenoid surface)			

**Fig. 3:**
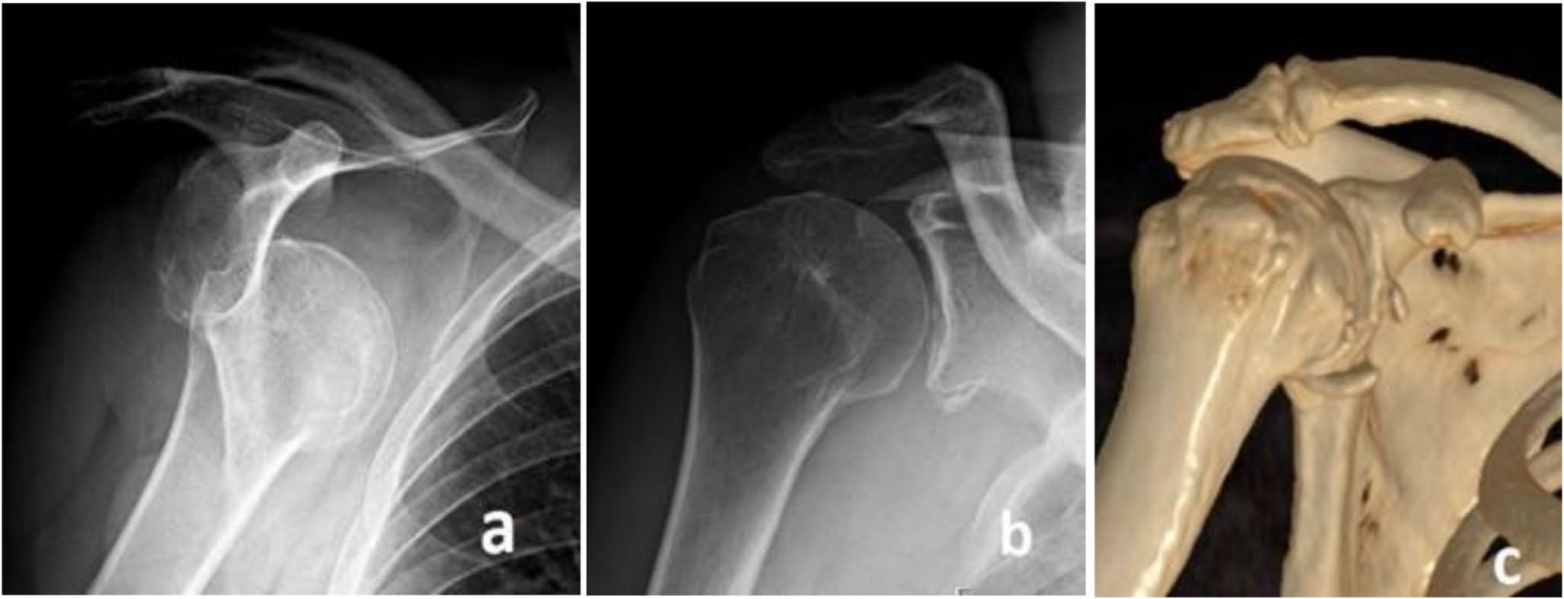
**a**: dislocated shoulder x-ray, **b**: post reduction Grashey view, **c**: 3D reconstruction CT scan

All patients were admitted through our Emergency Department, where a detailed clinical examination and radiological assessment, including AP and Grashey views, were conducted. In cases of shoulder dislocation, closed reduction was performed in the emergency room by an orthopedic surgeon. Imaging studies comprised radiography and computed tomography scans (CT scans) with surface-rendered 3-dimensional reconstructions [Fig. 3]. The head was considered centered on the glenoid based on the CT scan axial view with a subluxation index between 45–55% [28].

The shoulder was immobilized in a neutral rotation sling for four weeks, removed only for wrist and elbow mobilization three times a day for 10-minute sessions. After this period, passive mobilization was allowed on the scapular plane, avoiding external rotation. Once the complete range of motion was achieved, active mobilization with muscle strengthening and external rotations were permitted. Heavy strain on the shoulder was forbidden for three months after the trauma, and patients were allowed to return to pre-injury activities. Six months after the trauma, the return to sports activities was permitted.

### Follow-up evaluations

Clinical and radiographic (AP and Grashey views) follow-up was performed at 3 weeks, 6 weeks, 3 months, 6 months, 12 months, and 24 months. During clinical examinations, the range of motion (ROM) was assessed, including flexion, abduction, internal rotation (IR), and external rotation (ER). ER was measured using the elbow-on-the-table method [29], and IR was assessed as per Königshausen et al. [4]. similar to the Constant score, and scored on a 0–10 points scale. The ROM of the non-dislocated shoulder was also measured, so the differential ROM between injured and uninjured shoulder was evaluated as proposed by Wieser et al. [25]. Shoulder instability was clinically evaluated using the Apprehension test, Bony Apprehension test [30], Hyperabduction test [31] and Sulcus sign in ER1. The Rowe and UCLA scores were recorded at 24-month follow-up. Since the UCLA score includes a question on patient satisfaction, patients were divided into “satisfied” and “dissatisfied”. An UCLA score greater than or equal to 27 was considered as excellent, according to the literature [32, 33].

### Statistical analysis

The statistical analysis was conducted using SPSS statistics software (IBM: New York, United States) version 20.0. The Shapiro-Wilk test was used to assess the normality of distributions. Descriptive statistics (means, standard deviations, ranges as appropriate) were employed. Data were compared using Student’s T-test for parametric groups, Mann-Whitney U-test for unpaired non-parametric values, and the Wilcoxon signed-rank test for paired non-parametric data. P-values < 0.05 were considered statistically significant.

## Results

Twenty patients were evaluated in the study, comprising 12 males (60%) and 8 females (40%). The mean age at the time of dislocation was 66.65 ± 15.8 years old. The glenoid fracture size was small in 9 cases and medium in 11, based on the Kim classification [9]. Seven anteroinferior glenoid rim fractures were categorized as 1b type and 13 as 1c type, according to the Scheibel classification [10, 11]. The mean follow-up was 32.5 ± 7.53 months (range 24–48).

The average UCLA and Rowe scores at 24 months for the entire cohort were 32.15 ± 2.85 and 93.85 ± 2.19, respectively. Nineteen patients (95%) showed excellent UCLA values (≥ 27), and all patients (100%) were satisfied. The ROM analysis showed a mean flexion of 170° ± 18.71°, mean abduction of 170.77° ± 18.91°, mean ER1 of 61.92° ± 11.09°, and mean IR of 9.08 ± 1.04. The affected shoulder didn’t show significant reductions in ROM compared with the contralateral shoulder, except for a loss of ER (13.08° ± 7.51; *p* = 0.005) [Table 2]. No cases of post-traumatic instability were reported, and all patients were able to return to their work or sports activities.

**Table 2:** Comparison between the ROM of the dislocated shoulder with the ROM of the healthy shoulder

	Dislocated shoulder ROM (± SD)	Healthy shoulder ROM (± SD)	*P* value
Flexion	170° **±** 18.71°	179.23° **±** 2.77°	*p* = 0.126
Abduction	170.77° **±** 18.91°	179.23° **±** 2.77°	*p* = 0.253
External Rotation	61.92° **±** 11.09°	75° **±** 10.60°	***p*** = 0.005*
Internal Rotation	9.08° **±** 1.04°	9.08° **±** 2.78°	*p* = 0.152

No patients reported a positivity at the Sulcus sign in ER, and only 1 patient (5%) reported both apprehension, bony apprehension, and hyperabduction tests.

According to Kim calcification, the small-sized group had a mean fragment dimension of 8.27% ± 1.44, with a mean UCLA score of 32.14 ± 2.73, a Rowe score of 93.57 ± 2.44, and a mean ER1 of 12.14° ± 6.99°. The medium-sized group showed a mean fragment size of 21.13% ± 3.72%, with a mean UCLA score of 32.17 ± 3.25, a Rowe score of 94.16 ± 2.04, and a mean ER of 12.5° ± 6.89°. No statistical significance was found comparing the Kim small-sized group and Kim medium-sized group in terms of clinical scores (UCLA *p* = 0.989; Rowe *p* = 0.771; Delta ER1 *p* = 0.928) [Table 3].

**Table 3:** Comparison of clinical and functional outcome based on fragment size

Mean Values	Kim small-sized	Kim medium-sized	*P* value
UCLA score	32.14 ± 2.73	32.17 ± 3.25	*p* = 0.989
Rowe score	93.57 ± 2.44	94.17 ± 2.04	*p* = 0.7718
Delta ER	12.14 ± 6.99	12.5 ± 6.89	*p* = 0.928

UCLA score: University of California Los Angeles Score; Delta ER: Difference between dislocated shoulder external rotation and healthy shoulder external rotation

The Scheibel 1b-group had a mean UCLA score of 31.75 ± 2.66, a Rowe score of 94.37 ± 1.77, and a mean ER1 of 10.71° ± 8.86. The Scheibel 1c-group showed a mean UCLA score of 32.25 ± 3.59, a Rowe score of 92.5 ± 2.89, and a mean ER1 of 18.75° ± 8.54°. No statistical significance was found comparing Scheibel 1b and Scheibel 1c type of fragments (UCLA *p* = 0.215; Rowe *p* = 0.465; Delta ER1 *p* = 0.177) [Table 4].

**Table 4:** Comparison of clinical and functional outcome based on glenoid rim fragmentation

Mean Values	1B Scheibel type	1 C Scheibel type	*P* value
UCLA score	31.75 ± 2.66	32.25 ± 3.59	*p* = 0.215
Rowe score	94.37 ± 1.77	92.5 ± 2.89	*p* = 0.465
Delta ER	10.71 ± 8.86	18.75 ± 8.54	*p* = 0.177

UCLA score: University of California Los Angeles Score; Delta ER: Difference between dislocated shoulder external rotation and healthy shoulder external rotation

## Discussion

The treatment of anteroinferior glenoid rim fractures in the middle-aged population remains a subject of debate [11, 13, 25], specifically regarding the choice between surgical and non-operative approaches. Our study contributes valuable insights, demonstrating that the non-surgical approach in middle-aged patients yields excellent outcomes, with no recurrence (0%), favorable shoulder function, and high patient satisfaction. However, a notable reduction in external rotation (13.08° ± 7.51; *p* = 0.005) was observed. Analysis of clinical outcomes based on fragment size and quality (Scheibel classification [10, 11]) did not reveal significant differences. Nevertheless, patients with multifragmented rim fractures (1b type loss of ER = 10.71°; 1c type loss of ER = 18.75°) exhibited lower levels of external rotation. To our knowledge, this is the first study analyzing outcomes following anteroinferior glenoid rim fractures based on fragment characteristics.

In line with recent literature [3, 12, 34], our cohort demonstrated positive clinical outcomes, with average UCLA and Rowe scores of 32.15 ± 2.85 and 93.85 ± 2.19, respectively, comparable to or slightly higher than other studies. Spiegl et al. [3] reported 12 non-operatively treated anteroinferior glenoid rim fractures with an average Rowe score of 89 (range 63–100) at a 2-year follow-up. Königshausen et al. [34]. non-operatively treated 14 glenoid rim fractures with a mean fragment size of 5 mm, reporting an average Rowe score of 90.4 points (range 50–100 points). Shoulder range of motion (ROM) in our study, including a mean external rotation of 61.92° ± 11.09° and a loss of ER of 13.08° ± 7.51 compared to the healthy shoulder, was consistent with the literature [4, 12, 13, 25]. Wieser et al. [25] reported a mean ER of 60° ± 15° in their cohort of 48 patients with Ideberg 1B classification fractures following dislocation.

Maqueira et al. [13]. analyzed 14 patients with great-size fragment bony Bankart treated nonoperatively, resulting in no recurrences and high patient satisfaction, with 2 cases of ER deficit between 10 and 20°. These data suggested a good reliability of the conservative treatments in selected patients, providing optimal residual shoulder function. Notably, a similar loss of external rotation was reported in surgically treated patients. Porcellini et al. [1]. observed a mean external rotation loss of 9.7° ± 4.9° in a cohort of 48 patients with arthroscopically repaired bony Bankart fractures. It’s known [1, 21] that affecting the ER of approximately 10° did not impact the shoulder function, allowing most patients to return to sport and normal activity of daily living in line with the outcome we reported. For this reason, we feel confident in approaching the middle-aged population, who do not require high-performance shoulder function with non-surgical treatment.

Our study reported a low recurrence rate consistent with other studies in the middle-aged population [13, 25, 26, 34]. Waltenspül [26] et al. found no cases of recurrence at a 9-year follow-up after bony Bankart fracture, and Song et al. [6]. reported comparable recurrent dislocation rates between patients with and without fractures. Fujii et al. [35] performed a histologic analysis of 27 bony fragments and surrounding ligaments excised during the surgery, which revealed changes in the labral-ligamentous complex (8 out of 27 samples (29.6%) with extensive degeneration. Furthermore, Waltenspül et al. [26]. , highlighted a high healing potential of the bony fragment, emphasizing the importance of bone healing in preventing recurrence. According to our analysis, Salomonsson et al. [12]. , in a cohort of 51 patients, stated the importance of the bony fragment in this lesion, identifying age and rim fractures as the only factors lowering the risk of recurrence. They underlined the importance of bone healing in the post-dislocation process, resulting in a favorable prognostic factor.

Our findings suggest that ligamentous degeneration combined with bone healing results in a stiff bone-labral interface, leading to a functional stiffness and some loss of external rotation. Only one patient (5%) exhibited positivity in Apprehension, Bony Apprehension, and Hyperabduction tests, but interestingly, this patient did not experience any loss of range of motion. This observation supports the notion that a slight reduction in external rotation may act protectively against recurrence.

Comparing outcomes based on glenoid rim fracture size, we found no differences in clinical and functional scores, consistent with Königshausen et al.‘s findings [4] However, analyzing fragmentation according to Scheibel 1b and 1c types, patients with multifragmented glenoid rims showed greater external rotation loss compared to 1b type, although statistical significance wasn’t reached. While Scheibel et al. [11] reported a case series of 1c type bony Bankart fractures with similar Rowe scores, they identified fragments in the axillary pouch [Fig. 1B] in all cases, suggesting a potential explanation for the greater loss of external rotation due to the healing process. We believe that, since the vascularization is provided by the ligamentous-labral complex [35], the fragments remain vascularized and are able to heal [26]. So, the healing of a fragment in the axillary pouch could be the explanation for the greater loss of ER.

Despite limitations such as a small sample size, absence of CT scans for fragment healing assessment, and reliance on the best-fit circle method for quantifying glenoid bone loss [36], our study offers valuable insights. It is a prospective analysis comparing bony Bankart fractures based on fragment quality, contributing new perspectives to the conservative treatment of this injury.

## Conclusions

In conclusion, nonoperative treatment appears to be a viable and effective option for patients aged 50 and above with anteroinferior glenoid rim fractures resulting from glenohumeral dislocation. This approach yields favorable functional outcomes and carries a low risk of recurrence. While a mean reduction of 13° in external rotation (ER) was observed in the majority of patients, this may serve as a protective factor against post-traumatic instability. Notably, a more substantial loss of ER was noted in fractures characterized by fragmentation of the glenoid rim, specifically Scheibel type 1c bony Bankart fractures. These findings support the consideration of nonoperative management in this patient population, emphasizing the importance of personalized treatment strategies based on fracture characteristics and patient demographics.

## Data Availability

All data are available in the main text and tables. Additional information can be provided if solicited.
